# Strain-Controlled Thermal–Mechanical Fatigue Behavior and Microstructural Evolution Mechanism of the Novel Cr-Mo-V Hot-Work Die Steel

**DOI:** 10.3390/ma18020334

**Published:** 2025-01-13

**Authors:** Yasha Yuan, Yichou Lin, Wenyan Wang, Ruxing Shi, Chuan Wu, Pei Zhang, Lei Yao, Zhaocai Jie, Mengchao Wang, Jingpei Xie

**Affiliations:** 1 School of Materials Science and Engineering, Henan University of Science and Technology, Luoyang 471023, China; 15138753540@163.com (Y.Y.); xiejp@haust.edu.cn (J.X.); 2 Longmen Laboratory, Luoyang 471000, China; 15937917107@163.com (Y.L.); 15038647370@163.com (R.S.); z57007p@163.com (P.Z.); 3 Luo Yang CITIC HIC Casting and Forging Co., Ltd., Luoyang 471039, China; 4 Tianjin Key Laboratory of High Performance Precision Forming Manufacturing Technology and Equipment, National-Local Joint Engineering Laboratory of Intelligent Manufacturing Oriented Automobile Die & Mould, Tianjin University of Technology and Education, Tianjin 300222, China; wuchuan@tute.edu.cn (C.W.); yaolei15340829613@163.com (L.Y.); 13117974004@163.com (Z.J.); wmc1078185376@163.com (M.W.)

**Keywords:** hot-work die steel, strain control, thermal–mechanical fatigue, microstructural evolution, precipitated carbides, fatigue fracture

## Abstract

In response to the intensifying competition in the mold market and the increasingly stringent specifications of die forgings, the existing 55NiCrMoV7 (MES 1 steel) material can no longer meet the elevated demands of customers. Consequently, this study systematically optimizes the alloy composition of MES 1 steel by precisely adjusting the molybdenum (Mo) and vanadium (V) contents. The primary objective is to significantly enhance the microstructure and thermal–mechanical fatigue performance of the steel, thereby developing a high-performance, long-life hot working die steel designated as MES 2 steel. The thermal–mechanical fatigue (TMF) tests of two test steels were conducted in reverse mechanical strain control at 0.6% and 1.0% strain levels by a TMF servo-hydraulic testing system (MTS). The microstructures of the two steels were characterized using scanning electron microscopy (SEM), electron backscatter diffraction (EBSD), and transmission electron microscopy (TEM). The results indicate that throughout the entire thermomechanical fatigue cycle, both steels exhibit initial hardening during the low-temperature half-cycle (tension half-cycle) and subsequent continuous softening during the high-temperature half-cycle (compression half-cycle). Furthermore, under the same strain condition, the cumulative cyclic softening damage of MES 1 steel is more pronounced than that of the newly developed MES 2 steel. The number, width, and length of cracks in MES 2 steel are smaller than those in MES 1 steel, and the thermomechanical fatigue life of MES 2 steel is significantly longer than that of MES 1 steel. The microstructures show that the main precipitate phase in MES 1 steel is Cr-dominated rod-shaped carbide. It presents obvious coarsening and is prone to inducing stress concentration, thus facilitating crack initiation and propagation. The precipitate phase in MES 2 steel is mainly MC carbide containing Mo and V. It has a high thermal activation energy and is dispersed in the matrix in the form of particles, pinning dislocations and grain boundaries. This effectively delays the reduction in dislocation density and grain growth, thus contributing positively to the improvement in thermomechanical fatigue performance.

## 1. Introduction

Die steel is a type of steel used to manufacture dies such as cold stamping dies, hot forging dies, and die-casting dies. Due to the different uses and complex working conditions of various dies, the steel used for dies should have high hardness, strength, wear resistance, sufficient toughness, as well as high hardenability and other properties [1,2]. Research has shown that isothermal fatigue and thermomechanical fatigue failure are the main failure modes of hot work die steel [3,4]. At the same time, they are one of the most complex issues in terms of component fatigue damage, which involves various influencing factors such as temperature, load, oxidation, creep, and so on. In recent years, scholars have conducted in-depth research on isothermal fatigue (IF) and its relationship with thermal–mechanical fatigue (TMF) to explore their effects on the deformation behavior and damage mechanisms of metallic materials.

In terms of fatigue damage mechanisms, Yin et al. [5] conducted experiments on IF and TMF and found that the increase in temperature and thermal cycling can lead to the saturation of cyclic peak stress and a significant reduction in fatigue life. Their finding provides an important theoretical basis for understanding the effects of thermal cycling on materials. The damage evolution model and isothermal creep–fatigue model proposed by Xu et al. [6] were validated through tension hold experiments. Additionally, Lu et al. [7] explored the role of environmental damage in predicting fatigue life and found that environmental factors have a significantly greater impact on fatigue life than creep, proposing a unified fatigue model. Bartošák et al. [8] reported that the main failure modes in isothermal low-cycle fatigue and thermal–mechanical fatigue tests were interface debonding and intragranular crack propagation. Their damage model combines creep and fatigue damage while indirectly considering the effects of oxidation. Cao et al. [9] thermal–mechanical fatigue (TMF) tests and isothermal fatigue (IF) tests were conducted using thin-walled tubular specimens under strain-controlled conditions. The results of TMF tests showed a strong correlation between mechanical behavior and temperature cycling. Yin et al. [10] thermomechanical fatigue tests were performed on austenitic stainless steel in the temperature range of 250–400 °C. The results show that the increase in strain amplitude reduces fatigue life and enhances the impact of the phase angle. Wang et al. [11] proposed a new energy-based model for low-cycle fatigue (LCF) and TMF life prediction based on the hysteresis energy with strain rate modification, considering both fatigue and creep damages. The predicted results agree well with the experimental ones for the Al-Si piston alloy.

In addition, the study of fatigue damage mechanisms under specific loading conditions is also noteworthy. Zheng et al. [12] investigated the effect of high-cycle fatigue (HCF) loading on the crack propagation behavior of 316LN stainless steel during IF and TMF. They found that HCF loading significantly increased the crack propagation rates in IF and non-phase TMF tests, while its impact on phase TMF tests was minimal, mainly due to differences in the plastic zone at the crack tip. Wang et al. [13] showed that under different tensile hold times, fatigue-dominated intragranular cracks led to fractures in creep–IF, whereas in creep–TMF, the damage mechanism transitioned to creep–fatigue interaction with increasing hold time. Li et al. [14] noted that at a strain amplitude of approximately 0.27%, the lifetime curves of IF and TMF intersected, and the maximum stress was considered a key parameter for lifetime prediction. Meanwhile, Lin et al. [15] studied the effects of multiaxial loading on fatigue and found that non-proportional multiaxial loading significantly reduced the lifespan of IF and led to notable differences in damage mechanisms under different loading conditions. Wu et al. [16], based on the strain-controlled thermomechanical fatigue (TMF) experiments, concluded that under the in-phase (IP) and out-of-phase (OP) thermomechanical fatigue conditions, the stress–strain response curves of AISI H13 steel under different mechanical strain amplitudes show a similar evolution tendency.

The chemical composition significantly influences the microstructure and fatigue performance of hot work die steels, and proper adjustment can enhance toughness and fatigue resistance. Di et al. [17] reduced the vanadium content to minimize the formation of large-sized MC-type carbides and decreased the silicon content to promote cementite dissolution, leading to fine and uniformly distributed secondary carbides. These adjustments improved toughness, inhibited crack propagation, and delayed the coarsening of M_23_C_6_ carbides. Gong et al. [18] demonstrated that nickel dissolves into the matrix, forming lattice distortions that facilitate the dispersion of secondary carbides during tempering. This reduces grain boundary embrittlement and significantly enhances toughness. However, prolonged tempering can accelerate carbide aggregation, reducing high-temperature anti-softening properties. Gu et al. [19] revealed that nitrogen improves tempering stability by regulating the precipitation and coarsening behavior of M_3_C and M_23_C_6_ carbides. Nitrogen also enhances precipitation hardening and grain refinement strengthening, which account for approximately 70% of the total yield strength, with its effects becoming more pronounced over extended tempering periods. Zhang et al. [20] found that the addition of molybdenum (Mo) significantly refined the grain size in low-carbon Cr-Ni-Mo die steels and improved high-temperature oxidation resistance. Mo inhibited the inward diffusion of oxygen and the outward diffusion of iron, promoting the formation of chromium oxide. Additionally, molybdenum oxides contributed to the development of a protective oxide film, further enhancing oxidation resistance. Hong et al. [21] discovered that coarse vanadium-rich MC carbides at grain boundaries significantly impair the thermal fatigue performance of hot work die steels. By reducing the content of MC-forming elements and optimizing forging processes to dissolve MC carbides and eliminate segregation bands of vanadium and carbon, the formation of coarse MC carbides was effectively suppressed, leading to substantial improvements in the thermal fatigue resistance of steels such as SeAH-VD.

The thermal fatigue properties of die steel are influenced not only by the original microstructure prior to fracture but also significantly by the type, size, and distribution of carbides at the fracture site [22,23]. A finer grain size and a more homogeneous microstructure enhance strength and toughness in steel, thereby improving its fatigue performance. Finely dispersed carbides also significantly enhance the thermal fatigue resistance of die steel [24,25,26,27,28,29].

Yang et al. [30] prepared Co-based coatings on H13 steel using a laser deposition method to enhance its thermal fatigue performance. They found that early-stage thermal fatigue tests exhibited oxidation phenomena, and residual thermal stresses caused microcracks due to the separation of carbides from the matrix. These studies indicate that the strength, hardness, wear resistance, and fatigue resistance of hot work die steels are closely related to the size and distribution of dislocations and carbides. Dong et al. [31] conducted thermal–mechanical fatigue experiments on a hot work die steel under different strain amplitudes (0.7% to 1.1%). They found that the thermal–mechanical fatigue cycle softening process could be divided into three stages: initial instability, continuous softening, and failure. As the strain amplitude increased, the coarsening of martensite and carbides in the microstructure of the tested steel decreased, while the length and width of fatigue cracks increased. Chen et al. [32] studied the thermal–mechanical fatigue behavior of 4Cr5Mo3V hot work die steel using a strain-controlled approach to investigate the synergistic effects of cyclic temperature and mechanical loading. The results confirmed that as the mechanical strain amplitude increased, the cyclic softening degree of 4Cr5MoV3 steel also increased, leading to a reduction in fatigue life. They also pointed out that the decomposition of martensite, oxidation, reduced dislocation density, and carbide segregation were all contributing factors to the ultimate TMF failure.

Currently, research on hot working die steels is largely focused on the Uddholm self-restrained fatigue testing method due to limitations in equipment and technology [33]. However, studies on hot mechanical fatigue remain scarce, despite this aspect being more representative of the actual service conditions of hot working dies and offering more reliable theoretical support for understanding their fatigue failure characteristics.

Zhang et al. [34] demonstrated that 55NiCrMoV7 steel undergoes rapid initial softening during low-cycle fatigue, followed by progressive softening that does not reach saturation. This softening behavior is primarily driven by the interaction of cyclic plastic deformation and thermal loads, resulting in significant hardness reduction and changes in martensite diffraction peak width. When the fatigue temperature exceeds the tempering temperature, these effects become more pronounced, further limiting the material’s performance under high-temperature fatigue conditions. To address these challenges, this study employs a strain-controlled methodology to investigate an innovative material developed by our team in comparison with the widely used 55NiCrMoV7 steel. The research examines their thermal–mechanical fatigue behavior under varying temperature and mechanical loading conditions, focusing on cyclic stress–strain responses, cyclic hardening/softening behaviors, fatigue lifespans, microstructural evolution, and fracture characteristics. The findings provide valuable theoretical insights for developing high-strength, tough hot-working die steels capable of meeting the stringent demands of large-scale, high-end applications.

## 2. Experimental Materials and Methods

### 2.1. Experimental Materials

In response to those challenges mentioned in Refs. [35,36], our team has independently developed an improved variant of 55NiCrMoV7 specifically tailored for large-scale forging applications in molds. This modified alloy incorporates a high concentration of Mo and V-elements recognized for their strong carbide-forming capabilities, which facilitate the precipitation of fine dispersed MC- and M_2_C-type strengthening phases while enhancing the austenitizing temperature, thus inhibiting grain coarsening during heating processes and consequently improving both high-temperature strength as well as resilience against hot fatigue, which is essential for meeting the demanding operational environments typical in large mold applications.

The steel named MES 1 used in this study refers to 55NiCrMoV7, and MES 2 represents the new developed die steel by our team, which were melted in a vacuum induction furnace. The main chemical composition is shown in Table 1. The manufacturing process comprised the following steps: melting 50 kg of steel ingots in a vacuum induction furnace, subsequent annealing, removal of water risers, forging, post-forging heat treatment, and the production of the test steel. Thereafter, the test steel was machined into 30 × 30 × 300 mm specimens using cutting equipment for further analysis.

### 2.2. Heat Treatment Process

Two steel specimens, designated as MES 1 and MES 2, each measuring 30 × 30 × 300 mm, were placed in a box-type heat treatment furnace for the heat treatment process. To facilitate a comparison of their thermal–mechanical fatigue properties, the heat treatment parameters were initially adjusted to ensure that both specimens achieved the same hardness level. Table 2 provides detailed information on the heat treatment process parameters and the corresponding hardness values for both steels.

### 2.3. Thermal Fatigue Test

**Figure 1:**
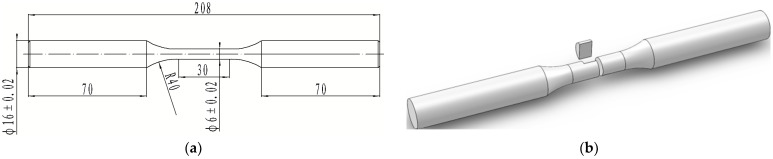
TMF sample size diagram and sampling schematic diagram: (**a**) dimensional drawing, unit: mm; (**b**) sampling diagram.

The thermal fatigue specimens were extracted from a 30 × 30 × 300 mm test bar in its heat-treated condition, as illustrated in Figure 1. The thermal–mechanical fatigue (TMF) tests were conducted using an MTS 809 hydraulic servo testing machine (Eden Prairie, MN, USA), which is capable of delivering a maximum axial load of 250 kN. The gauge length of the TMF specimen was heated using an electromagnetic induction coil, and the temperature was continuously monitored with a thermocouple attached to the gauge length. The temperature accuracy was maintained within ±3 °C, and the axial tensile and compressive mechanical strain was controlled to within 0.01% using an axial high-temperature ceramic extensometer. Specimen cooling was achieved using compressed air. All TMF tests were conducted under atmospheric conditions. Fatigue tests were strain-controlled, with strain ranges of −0.6% to 0 and −1% to 0, selected based on operational requirements. The cyclic temperature range for the TMF test was set between 400 and 600 °C, with each cycle lasting 2 min. The TMF test employed an out-of-phase and frequency-matched temperature and strain cycling (OP, Φ = 180°), where the maximum tensile strain coincided with the peak temperature. Both the temperature and strain cycling waveforms were triangular, as depicted in Figure 1a. All fatigue tests were conducted until specimen failure, and all equipment was computer-controlled with automatic data recording.

### 2.4. Microscopic Characterization

To observe and analyze the changes in microstructure near the fatigue fracture surface and the initiation and propagation mechanism of thermal fatigue cracks on the surface of the test steel, a molybdenum wire cutting machine was used to cut and sample the fractured sample along the axial direction, as shown in Figure 1b. After mechanical grinding, polishing, and 4% nitric acid alcohol corrosion, a metallographic sample was prepared. The surface crack state was photographed under a Zeiss 200MAT metallographic microscope (Oberkochen, Germany), and the fatigue fracture morphology and microstructure of the sample were observed under a Zeiss EVO18 scanning electron microscope (Oberkochen, Germany). The micro element analysis of the sample was carried out at 20 KV voltage using an X-MaxN spectrometer (Oxford, UK), including a point scan, line scan, and surface scan. To ensure shooting quality, the sample needs to be cleaned with anhydrous ethanol as a cleaning solution using ultrasonic waves for 30 min before shooting.

The EBSD samples were prepared using conventional metallographic techniques. Subsequently, the designated testing region underwent ion beam polishing using the JEOL IB-19530CP equipment equipped (Tokyo, Japan) with argon ion technology. The specific parameters utilized were an accelerating voltage of 8.0 kV and a polishing duration of 30 min. The JSM-7800F field emission scanning electron microscope was equipped (Tokyo, Japan) with an electron backscatter detector to analyze the grain size and crystal orientation of two types of steel after fatigue fracture under different strain conditions. TEM sample preparation was performed using a Gatan 691 PIPS ion thinning machine (Fremont, CA, USA). A 3 mm diameter circular sample was thinned at an initial angle of 6° for 2 h until a perforation appeared. The angle was then reduced to 3°, and the thinning continued for an additional 20 min. The acceleration voltage during the detection process is 30 kV, the working distance is 15 mm, the sample tilt angle is 70°, and the working step size is 60 nm.

## 3. Results

### 3.1. Fatigue Cycle Characteristic

**Figure 2:**
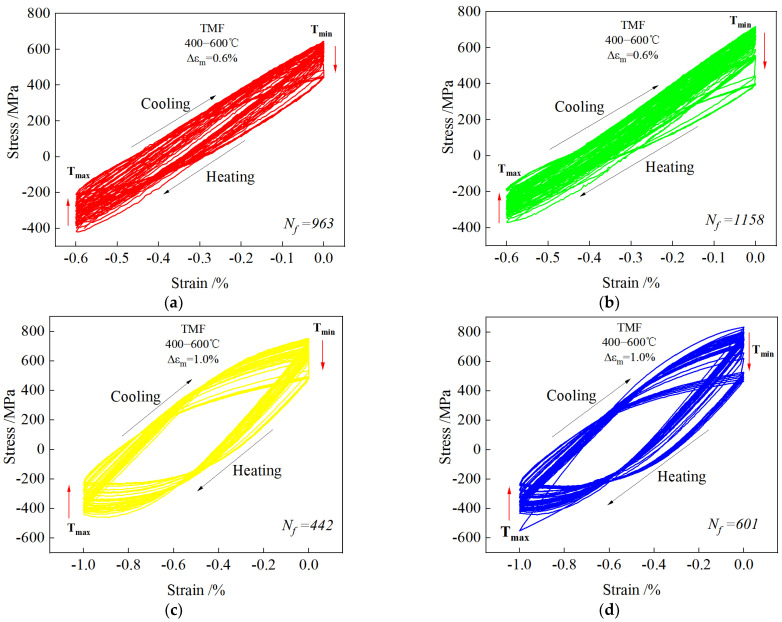
Stress–strain hysteresis curves under different mechanical strains. (**a**,**c**) MES 1 steel; (**b**,**d**) MES 3 steel.

**Figure 3:**
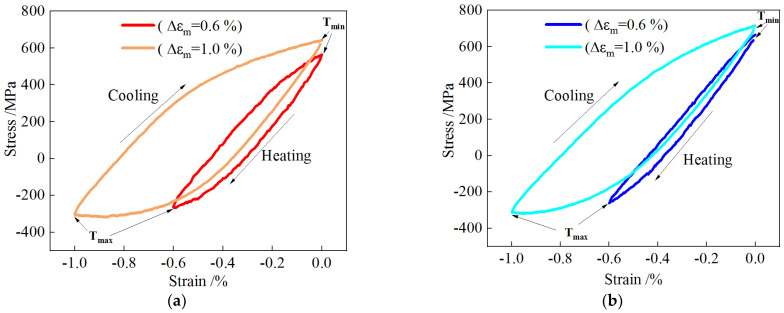
Hysteresis curves at half-life under different mechanical strain amplitudes (400–600 °C): (**a**) MES 1 steel; (**b**) MES 2 steel.

Figure 2 shows the stress–strain hysteresis loops for MES 1 and MES 2 steels during the TMF cycle at 400 to 600 °C and strains of −0.6% and −1.0%. As shown in Figure 3, it is evident that the temperature and mechanical strain exhibit asynchronous variations, with the temperature increasing during compression and decreasing during tension. Specifically, the temperature reaches its peak cycle value of 600 °C when the mechanical strain attains its minimum value of −0.6% or −1.0% (maximum compressive strain) within the cycle; conversely, when the mechanical strain returns to zero, the temperature drops to its lowest cycle value of 400 °C. The figure illustrates that the stress–strain hysteresis loop over the entire service life demonstrates cyclic softening under mechanically controlled strain conditions. This indicates that, as the cycle progresses, the peak stress of both steels diminishes with an increasing number of cycles (as indicated by the red arrows in the figure). This phenomenon is primarily attributed to the reduction in steel strength due to elevated temperatures during the thermomechanical fatigue (TMF) cycle, which leads to a decrease in the material’s resistance to plastic deformation. Under identical strain conditions, MES 2 steel exhibits a higher number of cycles compared to MES 1 steel, as depicted in Figure 2a,b, where Δε_m_ is 0.6%. The respective number of cycles for MES 1 steel and MES 2 steel is 963 and 1158 when Δε_m_ is 0.6%. Similarly, as shown in Figure 2c,d, where Δε_m_ is 1.0%, the number of cycles for MES 1 steel and MES 2 steel is 442 and 601, respectively. Furthermore, the peak stress of MES 2 steel exceeds that of MES 1 steel, primarily due to the higher strength of MES 2 steel at the same tensile temperature. In conclusion, under equivalent TMF conditions, MES 2 steel demonstrates superior fatigue strength and a longer fatigue life.

Figure 3 presents the half-life stress–strain hysteresis curves for the steels at different mechanical strain amplitudes, which reached a stable state. The area of the hysteresis loop represents the plastic strain energy absorbed by the steel [37]. As shown in Figure 3b, the hysteresis curve of MES 2 steel at a mechanical strain amplitude of −1.0% is wider and more diamond-shaped compared to the amplitude of −0.6%, with a significantly larger enclosed area, indicating that the energy loss increases and damage worsens as the mechanical strain amplitude increases. Figure 3a shows the stable half-life hysteresis curve of MES 1 steel, which is wider and encloses a larger area compared to MES 2 steel, demonstrating that MES 1 steel experiences greater energy loss and has weaker resistance to tension and compression.

### 3.2. Stress Analysis

**Figure 4:**
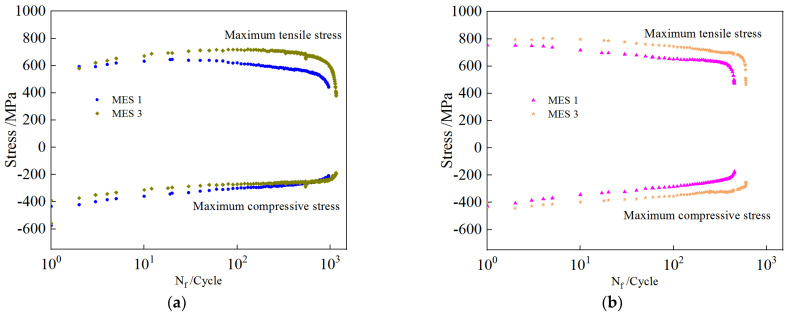
Cycle stress response curves under different mechanical strain: (**a**) Δε_m_ = 0.6%; (**b**) Δε_m_ = 1.0%.

Figure 4 illustrates the cyclic stress response curves for two steels subjected to varying strain amplitudes. The data reveal a consistent trend: during the low-temperature half-cycle (tension half-cycle), the cyclic stress response initially exhibits minor cyclic softening, which subsequently progresses into sustained cyclic softening. Conversely, throughout the high-temperature half-cycle (compression half-cycle), continuous cyclic softening is observed. This phenomenon could be attributed to the matrix’s cyclic softening, facilitated by dislocation climb and cross-slip-induced dislocation reorganization and annihilation at elevated temperatures, leading to the formation of dislocation loops and tangles [38]. In contrast, during the early stages of the low-temperature half-cycle, the accumulated dislocations do not sufficiently counteract the dislocation strengthening effect. Subsequently, the matrix undergoes continuous cyclic softening due to the synergistic effects of dislocation reorganization and annihilation [39], matrix recovery and recrystallization, and carbide growth. Furthermore, it is noteworthy that specimens subjected to varying strains demonstrate a rapid decline in cyclic stress prior to fracture. This phenomenon primarily results from the process of heat dissipation and the propagation of macroscopic cracks, which culminates in fracture following the initial formation of these cracks. From the curve, it is evident that in the TMF tests of MES 1 steel and MES 2 steel, the maximum tensile and compressive stresses exhibit asymmetry. However, both tensile and compressive stresses consistently decrease as the number of cycles increases, with the reduction being more pronounced during the tensile half-cycle. This suggests that both types of steel demonstrate cyclic softening and cumulative damage throughout the entire TMF process. The entire TMF cycle response process can be divided into the following three stages [40]:

(1) Adaptation stage: during the first few cycles, due to the sudden application of a non-isothermal, non-constant mechanical strain, the microstructure and cyclic deformation do not yet respond synchronously, usually requiring a few cycles of adaptation.
(2) Softening stage: This stage is the primary phase leading to cumulative damage during the entire cycle. During this phase, stress decreases continuously (cyclic softening), which is directly related to the evolution of the steel’s microstructure.
(3) Failure stage: This is the final stage of the entire fatigue test. At this point, cracks in the specimen have propagated to a significant depth, making them prone to further growth under external forces, leading to macroscopic cracking and eventual failure.

### 3.3. Microstructural Evolution

#### 3.3.1. Fatigue Performance Analysis Under Different Strains

**Figure 5:**
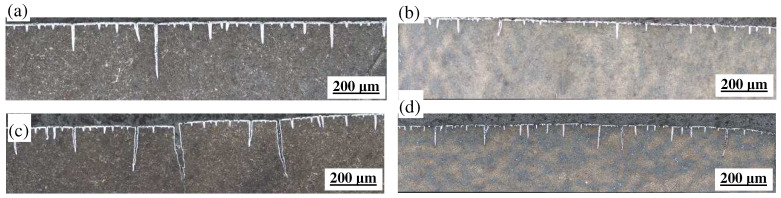
Thermal fatigue crack morphology of two types of steel under different strains: (**a**) MES 1 steel strain 0.6%; (**b**) MES 2 steel strain 0.6%; (**c**) MES 1 steel strain 1.0%; (**d**) MES 2 steel strain 1.0%.

Figure 5 illustrates the morphology of thermal fatigue cracks in two steels subjected to various strains within a cycling temperature range of 400–600 °C. To quantitatively evaluate the thermal fatigue properties of these steels, statistical analyses were conducted on the average crack length, maximum crack width, and maximum crack length under different strain conditions, as depicted in Figure 5 and detailed in Table 3. It is evident from Figure 5 that cracks initiate from the oxide film present on the specimen surface, which continues to grow alongside crack propagation. Both the surfaces where cracks propagate and their tips exhibit an oxide film of uniform thickness. When cycled at identical strain amplitudes, significant differences are observed in the surface crack morphology between the thermal–mechanical fatigue specimens of both steels, with notable variations in terms of the number, length, and width of cracks. Under low strain conditions (strain amplitude set at 0.6%), as shown in Figure 5a,b, MES 1 steel exhibits a greater quantity of cracks along with larger lengths and widths compared to MES 2 steel, which features shorter crack lengths, narrower widths, and predominantly fine cracks. The characteristics—number, length, and width—of surface-initiated cracks in MES 2 steel surpass those found in MES 1 steel; specifically, its average crack length is reduced by 25.7% relative to that of MES 1 steel, while its maximum crack width is narrowed by 24.3%, indicating superior resistance against thermal–mechanical fatigue crack initiation. As the strain amplitude increases to 1.0%, substantial changes occur within both steels’ thermal fatigue crack morphology; notably increased numbers of fatigue cracks appear on both MES 1 and MES 2 specimens—with similar quantities but distinct differences regarding their widths: compared to that measured for MES 1 steel’s maximum width being significantly wider by approximately 46.5%, while also exhibiting an average length reduction by about 40.1%. This suggests that MES 2 steel demonstrates enhanced performance when resisting the expansion associated with thermal–mechanical fatigue cracking phenomena. According to the above analysis, the initiation and propagation of the fatigue crack resistance of MES 2 steel is better than that of MES 1 steel under the condition of thermal–mechanical fatigue service.

**Figure 6:**
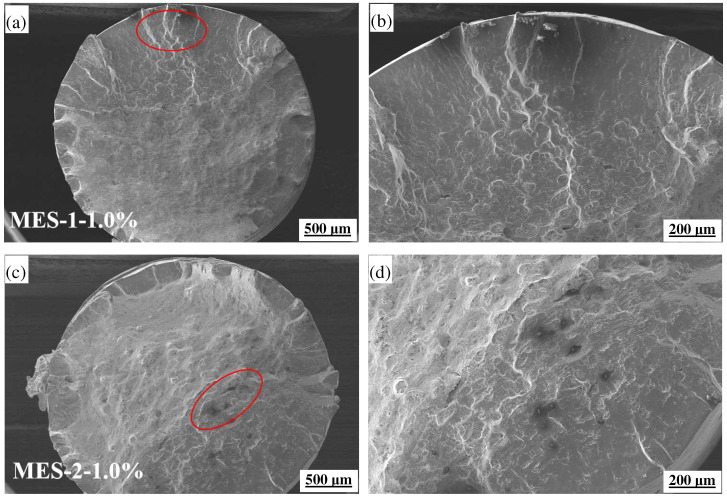
TMF macroscopic fracture morphology of MES 1 and MES 2 at 400–600 °C: (**a**,**b**) MES 1 steel-1.0%; (**c**,**d**) MES 2 steel-1.0%.

Figure 6 shows the cross-section macro morphology of MES 1 and MES 2 after thermal–mechanical fatigue cycles at 400–600 °C. Among them, Figure 6b,d are enlarged images of the red circles in Figure 6a and Figure 6c, respectively. It can be seen that there is no obvious boundary between the three regions of the fatigue fracture of MES 2. The crack initiation and fracture zones are very small, and the propagation zone occupies most of the fracture area. The fatigue fringes are more detailed, which indicates that the fatigue crack propagation rate is slower and reflects better fatigue resistance. In the fatigue fracture zone, MES 2 has a more dimpled structure, indicating that it has higher toughness. The dimples of MES 1 are less and shallow, indicating that it is more brittle in the final fracture stage. At the same time, the fatigue fractures of MES 1 and MES 2 in the figure have different degrees of oxidation, and the fracture surface of MES 2 shows a thinner oxide layer, which means that its high temperature oxidation resistance is stronger.

**Figure 7:**
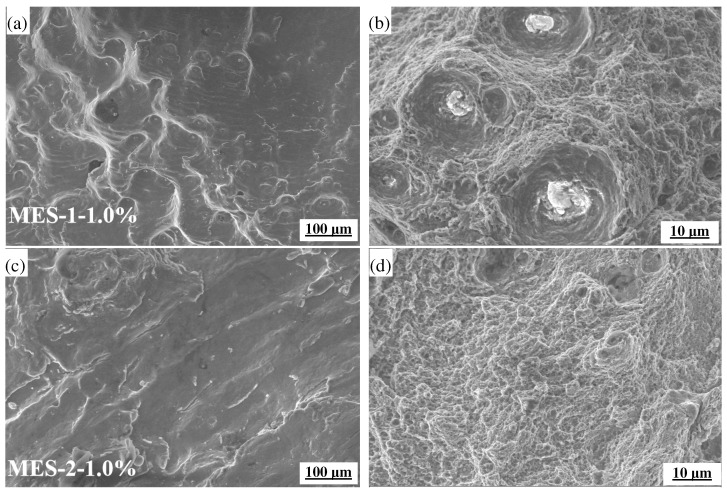
TMF fracture microstructure of MES 1 and MES 2 at 400–600 °C: (**a**,**b**) MES 1 steel-1.0%; (**c**,**d**) MES 2 steel-1.0%.

Figure 7 shows the thermo–mechanical fatigue fracture structure of MES 1 and MES 2 at 400–600 °C. The fatigue fracture zone is an important area to reflect the plastic or brittle fracture of the material [41]. Through further observation of the fracture zone, it was found that the instantaneous instability fracture zones of the two materials are basically similar. Similar to the fracture of the tensile test, there are more carbides and inclusions on the fracture. Of course, due to oxidation, there are also a large number of oxides. It can be observed that a more uniform and fine carbide distribution appears in MES 2, which helps to improve the fatigue performance of the material, because uniformly distributed carbides can disperse stress and hinder crack propagation [42]. Due to the higher content of strengthening elements (such as vanadium, molybdenum, or chromium) in MES 2, these elements can improve the creep resistance and fatigue resistance of materials under high-temperature conditions. However, the carbides of MES 1 are larger and unevenly distributed, which will lead to stress concentration and accelerate the initiation and propagation of fatigue cracks. Therefore, MES 2 shows strong fatigue resistance and a longer service life.

#### 3.3.2. The Impact of Thermal Fatigue on Tissues

**Figure 8:**
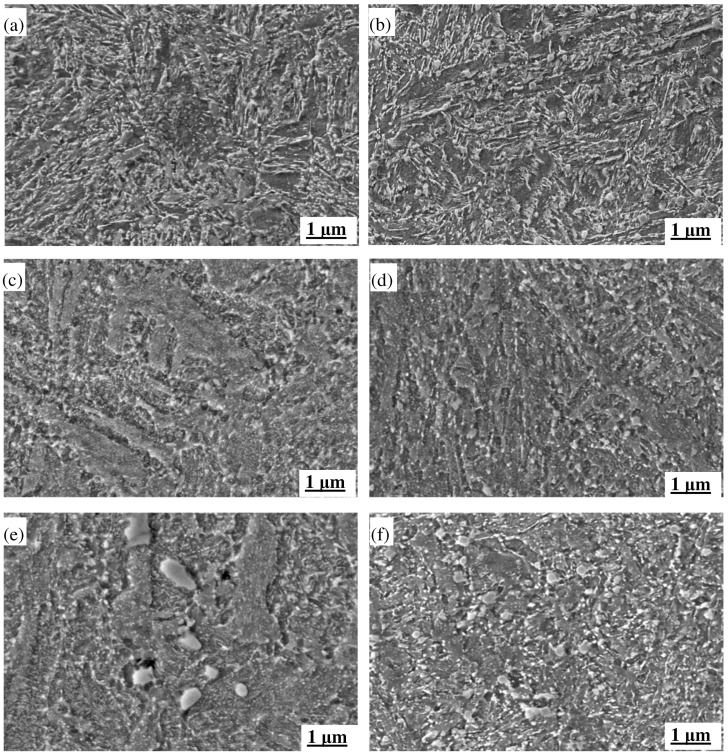
SEM images of two types of steel under different strains (temperature cycling 400–600 °C): (**a**) MES 1 steel original structure; (**b**) MES 2 steel original organization; (**c**) MES 1 steel strain 0.6%; (**d**) MES 2 steel strain 0.6%; (**e**) MES 1 steel strain 1.0%; (**f**) MES 2 steel strain 1.0%.

During thermal fatigue testing, the microstructures of MES 1 and MES 2 steels underwent significant changes, which directly influenced their thermal fatigue properties. As shown in Figure 8, the initial microstructures of both steels consist of tempered martensites and carbides, with MES 2 steel exhibiting finer bundles of tempered martensite platelets. At a 0.6% strain, MES 1 steel experienced a notable increase in the width of martensite platelets and a significant growth in carbide size. These changes hindered dislocation movement, leading to increased energy dissipation. In contrast, MES 2 steel showed only slight coarsening of the tempered martensite and a moderate increase in both the quantity and size of carbides, but these changes were more controlled, helping to reduce energy loss.

At a 1.0% strain, MES 1 steel displayed clear signs of recrystallization, with a blurred morphology of martensite platelets, enlarged grains, and further growth of carbide particles, leading to an increase in energy dissipation. In contrast, MES 2 steel maintained its fine martensite platelets, with only minor coarsening of some carbides. The overall microstructure remained dominated by fine, dispersed carbides, and the less extensive recrystallization in MES 2 steel helped significantly reduce the increase in energy loss.

The superior thermal fatigue properties of MES 2 steel can be attributed to its more stable microstructure, which consists primarily of tempered martensites interspersed with fine carbide particles. The fine and uniformly distributed carbides in MES 2 steel provide high thermal stability, preventing stress concentration and crack propagation, thus effectively reducing energy loss during thermal cycling. In contrast, the larger carbides and more extensive recrystallization in MES 1 steel lead to greater energy dissipation, which adversely affects its thermal fatigue performance.

## 4. Discussion

### 4.1. Effects of Mo and V Carbides on the Microstructural Evolution

**Figure 9:**
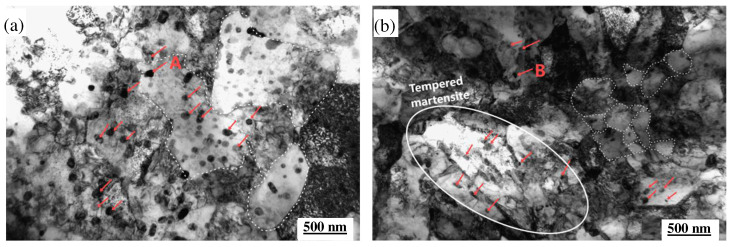

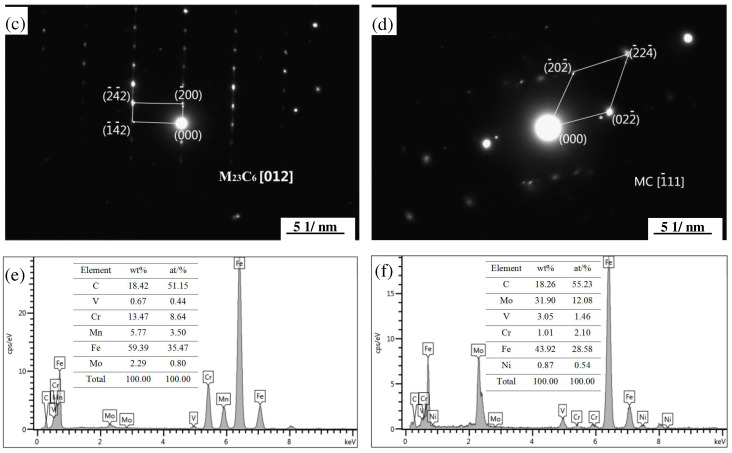
TEM micrographs, EDS elements image, and diffraction patterns of two steel types under 1.0% strain (temperature cycling at 400–600 °C): (**a**) MES 1 steel; (**b**) MES 2 steel; (**c**) electron diffraction pattern at arrow A in (**a**); (**d**) electron diffraction pattern at arrow B in (**b**); (**e**) EDS analysis at arrow A in (**a**); (**f**) EDS analysis at arrow B in (**b**).

Figure 9 illustrates the TEM images of two steel variants subjected to hot mechanical fatigue at a strain level of 1.0% over a temperature range of 400 to 600 °C. As depicted in Figure 9a,b, both steels exhibit distinct degrees of recovery and recrystallization following fatigue exposure. In MES 1 steel, the microstructure reveals a pronounced recrystallization devoid of discernible martensitic features; the recrystallized grains have significantly enlarged, accompanied by large carbides, as shown in Figure 9a. In contrast, MES 2 steel maintains its lamellar martensitic characteristics with a high density of dislocations throughout its microstructure, exhibiting only localized dynamic recrystallization that results in relatively fine grain sizes along with finely dispersed carbides, as illustrated in Figure 9b. To further elucidate the types of carbides present within these steels, EDS elemental analysis and electron diffraction patterns were conducted on the carbides indicated by red arrows in Figure 9a,b, and detailed further in Figure 9c–f. The analysis presented in Figure 9c indicates that the precipitates identified in MES 1 steel are M_23_C_6_ carbides. EDS spectrum analysis displayed in Figure 9e confirms that this carbide type predominantly comprises Cr. Similarly, for MES 2 steel, the precipitates primarily consist of Mo- and V-containing MC-type carbides, as demonstrated in Figure 9f.

In comparison to MES 1, MES 2 demonstrates significantly higher concentrations of Mo and V, as well as an elevated quenching temperature relative to MES 1. This enhancement promotes the dissolution of additional alloying elements within the matrix, thereby increasing the decomposition temperature and effectively compromising the stability of the α phase matrix. Importantly, while the carbon content in MES 2 remains unchanged compared to that in MES 1, the increased levels of Mo and V—both highly effective carbide-forming elements—result in their preferential occupation of carbon sites during annealing, leading to the formation of relatively fine carbides. Furthermore, Mn and Cr are incorporated into the matrix to provide solute strengthening effects. Simultaneously, Mo and V interact with C to precipitate as carbides, predominantly resulting in MC-type carbides within MES 2 steel [43]; these exhibit semi-coherency with the matrix and possess a comparatively high thermal activation energy that renders them less prone to growth. In contrast, MES 1 steel contains lower levels of Mo and V, with Cr being predominant. Chromium readily forms M_7_C_3_ or M_23_C_6_ carbides with C, which lack coherency with the matrix and have a reduced thermal activation energy. These larger carbides are more susceptible to growth under fluctuations in temperature and stress conditions [44]; consequently, they can generate stress concentrations that may initiate cracks during thermal fatigue—a key factor contributing to enhanced thermal fatigue performance observed in MES 2 steel.

### 4.2. Effects of Microstructural Evolution on the Fatigue Strength and Life

**Figure 10:**
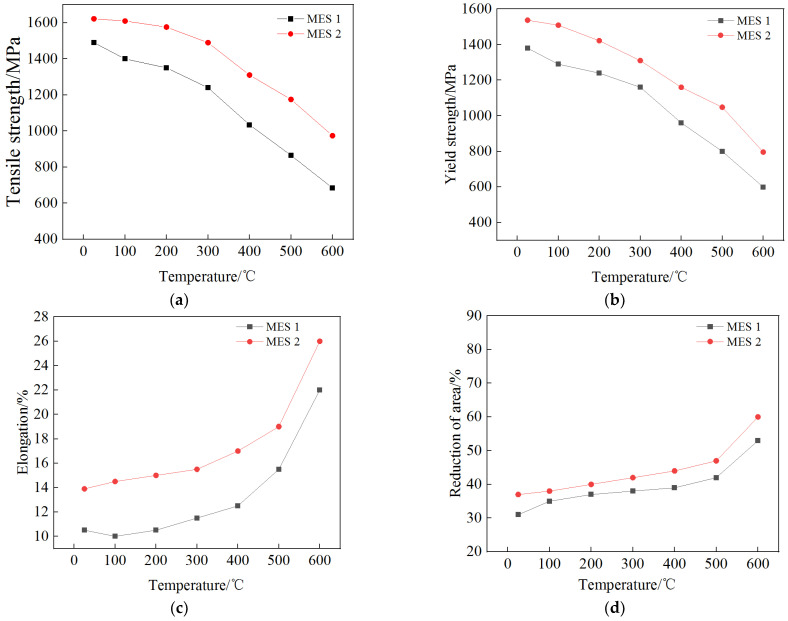
Comparison of high-temperature strength and ductility of two steel alloys. (**a**) Ultimate tensile strength; (**b**) 0.2% yield strength; (**c**) elongation; (**d**) reduction in area.

**Figure 11:**
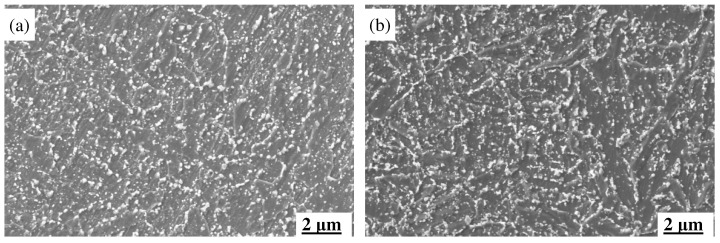
Microstructure of post-forging heat treatment: (**a**) MES 1 steel; (**b**) MES 2 steel.

**Figure 12:**
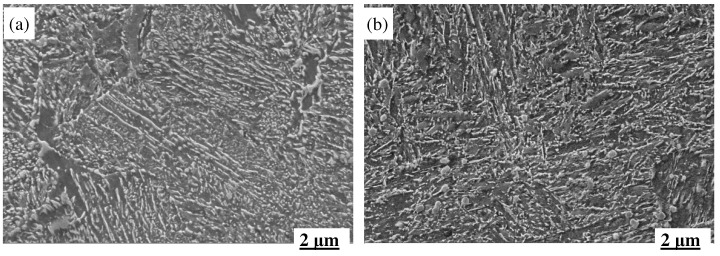
SEM images of the two steels after the QTHT: (**a**) MES 1 steel; (**b**) MES 2 steel.

Figure 10 depicts the hot-temperature strength and ductility of the two steels, which indicates that the MES 1 steel exhibits better comprehensive properties than the MES 2 steel, mainly due to the homogeneous microstructure formed in the former during the thermo–mechanical processing, as shown in Figure 11, it can be observed that few differences can be discerned from the metallographic micrographs of the two steels after the forging process. The main aim of the forging procedure is to mechanically crush the huge cast grains to obtain homogeneously fined grains. Subsequent to the forging process, a supplementary heat treatment is implemented to mitigate residual stresses, enhance microstructural stability, and condition the material for subsequent quenching and tempering. Therefore, there is no direct relation between the forged microstructure and the final properties of the steels. Figure 12 shows the SEM micrographs of the two steels after the quenched–tempered heat treatment (QTHT), which are totally different from the forged microstructure; moreover, obvious differences exist between the two micrographs. As depicted in Figure 12, there are significant disparities in the size and distribution morphology of the carbide precipitates between the two steels. The carbides formed in MES 1 steel predominantly display a striped morphology characterized by distinct directional alignment, with both the width and length of these stripes surpassing those illustrated in the right panel. Conversely, the carbides present in MES 2 steel are fine and uniformly distributed, exhibiting dimensions that are markedly smaller than those observed in MES 1 steel. The fine and uniformly distributed carbides in MES2 steel effectively suppress grain boundary migration under cyclic loading. Specifically, the Mo- and V-containing carbides act as pinning agents, obstructing the movement of grain boundaries during plastic deformation. Due to their small size and high number density, these carbides provide a more stable and effective pinning effect under cyclic stress. In contrast, the larger and more sparsely distributed carbides in MES1 steel offer weaker pinning, making the grain boundaries more susceptible to migration under cyclic loading. This inhibition of grain boundary migration contributes to the improved fatigue resistance of MES2 steel, effectively reducing the likelihood of crack initiation and propagation.

**Figure 13:**
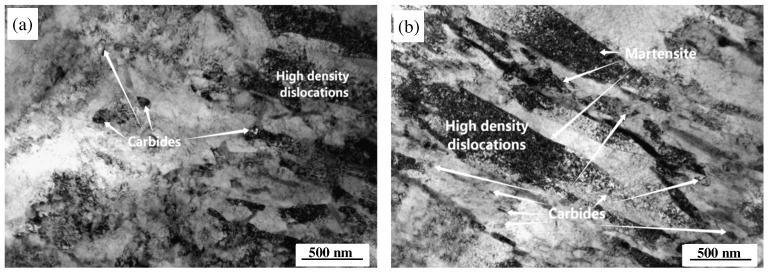
TEM images of the two steels after the QTHT: (**a**) MES 1 steel; (**b**) MES 2 steel.

To further explore the difference, the TEM images of the two steels after the QTHT process are given in Figure 13. It can be seen that the microstructures of the two steels after QTHT mainly consist of martensite and precipitated carbides. In MES 1 steel, a considerable fraction of the regions exhibits marked dynamic recrystallization, with the features of martensitic platelets being faintly discernible. The presence of high-density dislocation zones is limited, accompanied by sporadic, larger, irregularly shaped precipitated carbides dispersed throughout the matrix. In contrast, MES 2 steel displays a more pronounced morphology of platelet martensite, characterized by a significant density of dislocations within its structure. The precipitated carbides in this instance are relatively fine and uniformly distributed across the matrix, thereby providing a solid organizational basis for improved thermal fatigue resistance. Based on the above analysis, it is easily understood that the MES 1 steel has comprehensive properties superior to the MES 2 steel.

**Figure 14:**
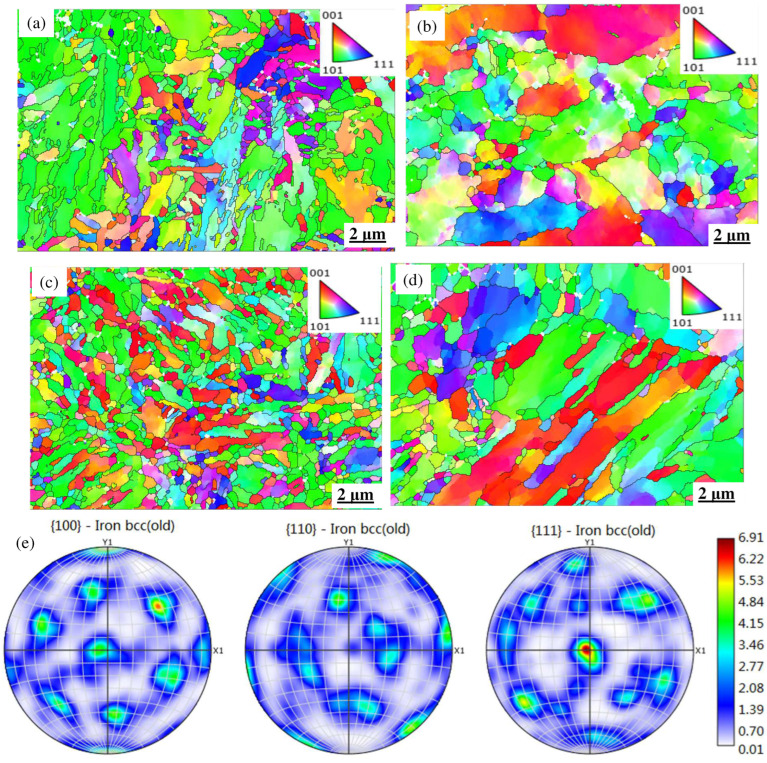
The reverse polarization curves of the two steel types at varying strains: (**a**) MES 1 steel (ε = 0.6%); (**b**) MES 2 steel (ε = 0.6%); (**c**) MES 1 steel (ε = 1.0%); (**d**) MES 2 steel (ε = 1.0%); (**e**) pole figure.

Figure 14 shows the inverse pole figures of the two steels parallel to different directions. It is clearly seen that the microstructural morphology of the MES 1 steel is completely different from that of MES 2. In Figure 14a,b, where the TMF condition is at a cycle temperature of 400 to 600 °C with a straining of 0.6%, the MES 1 alloy consists of many lath-tempered martensites with different thicknesses, lengths, and orientations, among which some equiaxed fined second-phase particles are homogeneously distributed, while the MES 2 alloy displays an obvious coarsening characteristic and some new recrystallized grains appear around the matrix boundaries. Clearly, a more heterogeneous deformation is present in the MES 2 alloy because of a gradient orientation in some coarsening grains. For the MES 1 alloy, a more tempered martensite has the <101> slipping direction parallel to the deformed direction (X1), while a preferred direction is commonly concentrated between the <101> and <001> direction. As the strain is increased to 1.0%, as shown in Figure 14c,d, a more short-rod-like tempered martensite appears in the MES 1 alloy, and a preferred orientation still belongs to the <101> direction. Moreover, some fine recrystallized grains with the <101> orientation are observed around the martensite boundaries. However, for the MES 2 alloy, the strain seemly imposes a significant effect on the morphological evolution. Clearly, most of the coarsening martensites in low strains have been replaced by the wide lath structure, and the orientation is relatively random; thus, no obvious texture can be observed for the MES 2 alloy under this condition, as shown in Figure 14e.

**Figure 15:**
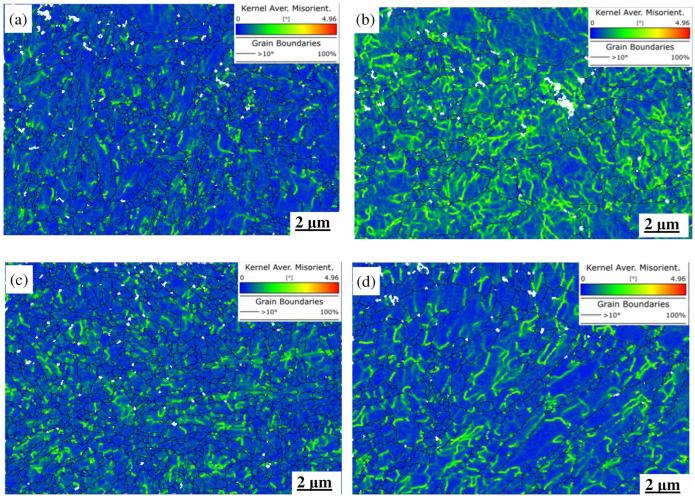
KAM diagram of two kinds of steel under different strains. (**a**) MES 1 steel (ε = 0.6%); (**b**) MES 2 steel (ε = 0.6%); (**c**) MES 1 steel (ε = 1.0%); (**d**) MES 2 steel (ε = 1.0%).

The corresponding Kernel Average Misorientation (KAM) maps are shown in Figure 15, which indicates that the KAM characteristics are different for the two steels. In the MES 1 alloy, as shown in Figure 15a,b, most of the KAM values homogeneously concentrate near the grain boundary zones, and some higher KAM values occasionally appear within the grains. In contrast, the distribution of KAM values in the MES 2 alloy exhibits a difference pattern in that most of the high KAM values appear within the grains, indicating a significant deformation gradient in these grains. As the strain is increased to 1.0%, as shown in Figure 15c, the KAM diagram of the MES 1 alloy seemingly changes a little compared with that in Figure 15a; only the KAM density is increased but its distribution is still in the boundary zones. However, for the MES 2 alloy, as shown in Figure 15d, the KAM density is reduced because of the decrease in grain areas, but the higher KAM values still concentrate within the grains, meaning that the non-uniform plastic deformation in the grain-scale is not vanished.

It can be found from the TMF tests that both the fatigue strength and the cycle of the MES 1 steel are better than those of MES 2, which means that the former alloy possesses the good ability of suppressing the crack initiation and propagation. The reasons behind it can be distinctly explained by Figure 14 and Figure 15. From these images, it is observed that the microstructural features, including the morphology, orientation, texture, and KAM values, of the MES 1 alloy are clearly different from those of the MES 2 alloy. For the MES 1 alloy, its microstructural structure consists of a heterogeneous lath, short-rod-like, and fine equiaxed grains; thus, it contains a high grain boundary area as well as high resistance of crack propagation. In addition, the alloy has a relatively homogeneous deformation in the grain-scale. Therefore, it is difficult for new cracks to initiate and propagate in this alloy and naturally a good anti-fatigue ability can be observed for the MES 1 alloy. In comparison, the anti-fatigue ability of the MES 2 alloy is inferior because of its simple homogeneous structure, lower grain boundary area and crack propagation resistance, and non-uniform deform in the grain-scale. Based on these factors, some micro-cracks are easy to initiate and propagate in this alloy subjected to the TMF tests, finally resulting in a lower strength and shorter fatigue life.

### 4.3. Prediction of Thermomechanical Fatigue Life

The factors affecting the fatigue life of mold steel include chemical composition, microstructure, internal defects, precipitate distribution, and matrix properties like toughness and strength. Current research on hot fatigue life in hot-working mold steel primarily uses the Manson–Coffin equation, which models hot fatigue as low-cycle fatigue. Failure typically occurs within 10,000 thermal cycles due to low-plastic-strain cycles, where the plastic strain amplitude (*ε_p_*) and the number of cycles to failure (*N_F_*) follow the Manson–Coffin relationship (Equation (1)).

(1) $$N_{F}^{n}\cdot \varepsilon_{p}=k\varepsilon_{f}$$

In this equation, *ε_p_* is the plastic strain amplitude per cycle; *n* and *k* are constants, where n is typically assigned a value of 0.5 in low-cycle fatigue scenarios. *ε_f_* denotes the true strain at the point of fatigue failure of the specimen, which can be determined from the measured cross-sectional reduction ratio (ψ) obtained through tensile testing, that is the following:

ε_f_ = −ln(1 − ψ) (2)

In the thermal fatigue process, the thermal strain is largely converted into two forms of mechanical strain: elastic and plastic. The plastic strain represents the amount of plastic deformation in each fatigue cycle, which can be mathematically expressed as follows:

(3) $$\varepsilon_{p}=\alpha \Delta T-\varepsilon_{c1}-\varepsilon_{c2}-\varepsilon_{T}$$

Among these parameters: *α* is the coefficient of thermal expansion; ΔT is the temperature cycle difference; and *α*ΔT is the total thermal strain.

Substituting Equation (3) into Equation (1) yields the model for predicting the thermal fatigue life of the heat engine as follows:

(4) $$N_{F}={\left(\frac{C\varepsilon_{f}}{\alpha \Delta T-\frac{(1-\nu_{1})\sigma_{1}}{E_{1}}-\frac{(1-\nu_{2})\sigma_{2}}{E_{2}}-\varepsilon_{T}}\right)}^{1/n}$$

In the actual service conditions of hot working die steel, the primary criterion for assessing its failure is the quantity and depth of fatigue cracks, rather than the occurrence of fatigue failure in the mold. Consequently, the precision of predicting the fatigue life of hot working die steel using a formula is limited. Nevertheless, it remains feasible to employ a formula to compare the hot fatigue life of different steels. Therefore, this chapter does not merely compute the fatigue life of MES 1 and MES 2 steels; instead, it utilizes Equation (4) to evaluate and compare their hot-mechanical fatigue life. To simplify the model, the elastic modulus E and Poisson’s ratio v are assumed to be constant and independent of temperature in this chapter. Equation (4) can thus be simplified as follows:

(5) $$N_{F}={\left(\frac{C\varepsilon_{f}}{\alpha \Delta T-\frac{\left(1-\nu_{1})(\sigma_{1}+\sigma_{2}\right)}{E_{1}}-\varepsilon_{T}}\right)}^{1/n}$$

Through the testing process, the thermal expansion coefficients of MES 1 and MES 2 steels at 600 °C were determined to be 1.41 × 10^−5^ and 1.37 × 10^−5^, respectively. Consequently, the αΔT value in Equation (5) remains essentially consistent. Furthermore, as illustrated in Figure 10, the elongation at fracture for the heat-treated MES 1 steel and MES 3 steel specimens was 10.7% and 13.9%, respectively. These results suggest that the strain value at fatigue fracture for MES 2 steel is significantly higher than that of MES 1 steel. Additionally, calculations conducted using JMatPro software (V13.2) indicated that the Poisson’s ratios and moduli of elasticity for MES 1 and MES 2 steels are comparable. Assuming identical residual strain values for MES 1 and MES 2 steels during both heating and cooling processes, Equation (5) can be further decomposed as follows:

(6) $$\left\{\begin{array}{c} \left(\frac{1}{N_{F}}{)}^{n}=A-B(\sigma_{1}+\sigma_{2}\right)\\ A=\frac{\alpha \Delta T-\varepsilon_{T}}{C\varepsilon_{f}}>0\\ B=\frac{1-\nu_{1}}{C\varepsilon_{f}E}>0 \end{array}\right.$$

Equation (6) shows that the stress values at the upper and lower limit temperatures during thermal fatigue testing are critical for assessing the thermal fatigue life of the experimental steel. Higher *σ*_1_ and *σ*_2_ values extend the thermal fatigue life. In this chapter, the upper and lower limit temperatures for the heat engine’s thermal fatigue testing are 600 °C and 400 °C, respectively. Figure 10 illustrates that the tensile strengths of MES 1 and MES 2 steels at 400 °C are 1034 MPa and 1310 MPa, respectively, and at 600 °C, they are 684 MPa and 994 MPa, respectively. These data show that MES 2 steel has superior high-temperature strength and higher peak stress values at the point of rupture compared to MES 1 steel. Thus, the total stress values for MES 2 steel in the thermal fatigue cycle are significantly higher than those for MES 1 steel. Comparative analysis of the equations confirms that MES 2 steel has a longer thermal fatigue life, consistent with the results of the thermal–mechanical fatigue tests.

## 5. Conclusions

The thermal–mechanical fatigue behavior of two types of steel within the temperature range of 400–600 °C was investigated through thermal–mechanical fatigue testing. The primary conclusions are as follows:

(1) In the tensile-compressive strain control mode (400–600 °C), MES 1 steel and MES 2 steel had thermal–mechanical fatigue lives of 963 cycles and 1158 cycles, respectively, at a 0.6% strain. At a 1.0% strain, their fatigue lives decreased to 442 cycles and 601 cycles. This shows that MES 2 steel has a notably higher fatigue life than MES 1 steel under the same strain.
(2) Both of the MES 1 and MES 2 steels exhibit thermal fatigue cracks under the same strain at a temperature cycle of 400 to 600 °C. However, the width and length of these cracks in the MES 2 steel are smaller than those in MES 1 steel. Moreover, the MES 2 steel shows better resistance to crack propagation.
(3) The martensitic plate-like morphology in MES 1 steel is gradually replaced by the recrystallized and recovery microstructure with straining, while the MES 2 steel exhibits a noticeable structural stability, still remaining a high density of dislocations and only localized recovery.
(4) The coarsened Cr-rich rod-shaped carbides are dominant in the MES 1 steel, causing the stress concentration and promoting the crack initiation and propagation. In contrast, the thermally stable fined Mo- and V-containing MC-type carbides are uniformly dispersed in the MES 2 steel, which effectively pin the dislocation motion and the grain boundary migration, thereby improving the hot mechanical fatigue strength and life.
(5) The TMF cycle includes adaptation, softening, and failure stages, with damage mainly in the softening stage. As the mechanical strain amplitude increases, MES 1 steel shows intensified cyclic softening, while MES 2 exhibits superior fatigue resistance due to its finer, more uniform carbide distribution, enhancing its fatigue strength and life.
(6) The tensile strengths of MES 1 steel and MES 2 steel are 1034 MPa and 1310 MPa, respectively, at 400 °C, and 684 MPa and 994 MPa, respectively, at 600 °C. Both the room temperature and high-temperature strengths of MES 2 steel are higher than those of MES 1 steel. This leads to a significantly prolonged thermal–mechanical fatigue life for MES 2 steel, which is consistent with the findings of the test samples. This conclusion provides a theoretical framework for predicting the thermal fatigue life of this type of large-scale die steel over its entire service period.

## Figures and Tables

**Table 1:** Chemical compositions of the tested steel (weight/%).

Element	C	Si	Mn	S	P	Cr	Ni	Mo	V	Fe
MES 1	0.56	0.20	0.81	0.0010	0.0065	1.17	1.75	0.52	0.10	Bal.
MES 2	0.56	0.20	0.83	0.0063	0.0046	1.20	1.73	1.09	0.71	Bal.

**Table 2:** Heat treatment process parameters and corresponding hardness values of thermomechanical fatigue specimens.

Steel Type	Quenching Temperature-Holding Time	Cooling Method	Tempering Temperature-Time-Frequency	HRC
MES 1	850 °C-1.5 h	oil cooling	570 °C-3 h-2	42.0
MES 2	920 °C-1.5 h	oil cooling	620 °C-3 h-2	42.6

**Table 3:** Crack statistics of two types of steel under different strains.

Strain Amplitude/%	MES 1 Steel			MES 2 Steel		
	Average Length of Cracks/μm	Maximum Crack Width/μm	Maximum Length of Crack/μm	Average Length of Cracks/μm	Maximum Crack Width/μm	Maximum Length of Crack/μm
0.6	66.7	33.42	496.76	49.56	25.29	241.81
1.0	153.25	61.09	626.62	91.77	32.70	384.64

## Data Availability

The original contributions presented in this study are included in the article. Further inquiries can be directed to the corresponding author.
